# Tetra­aqua­bis­(*N*,*N*-dimethyl­formamide-κ*O*)zinc(II) bis­[(2-{3-[2-(carboxyl­ato­meth­oxy-κ^2^ *O*,*O*′)phen­yl]pyrazol-1-yl-κ*N* ^2^}acetato-κ*O*)chloridozincate(II)]

**DOI:** 10.1107/S1600536812023045

**Published:** 2012-05-26

**Authors:** Jie Yang, Lei Shen, Cheng Ji, Xiao-Feng Shen, Gao-Weng Yang

**Affiliations:** a Department of Chemistry & Materials Engineering, Jiangsu Laboratory of Advanced Functional Materials, Changshu Institute of Technology, Changshu 215500, Jiangsu, People’s Republic of China

## Abstract

The asymmetric unit of the title compound, [Zn(C_3_H_7_NO)_2_(H_2_O)_4_][Zn(C_13_H_10_N_2_O_5_)Cl]_2_, is composed of a single anion and half a cation. The Zn^II^ atom in the monoanion has a distorted triganol–pyramidal geometry, being coordinated by three O atoms and one N atom from one 2-{3-[2-(carboxyl­ato­meth­oxy)phen­yl]pyrazol-1-yl}acetate ligand and one Cl atom. In the dication, the Zn^II^ atom is located on an inversion center and is coordinated by six O atoms in a slightly distorted octa­hedral geometry. In the crystal, the ions are linked by O—H⋯O hydrogen bonds, forming a two-dimensional network lying parallel to the *ab* plane. There are also C—H⋯O and C—H⋯Cl inter­actions present, which lead to the formation of a three-dimensional structure.

## Related literature

For potential applications of pyrazole derivatives in advanced materials, see: Su *et al.* (2000 ▶); Tong *et al.* (2003 ▶). For the τ-descriptor of penta-coordinated metal atoms, see: Addison *et al.* (1984 ▶).
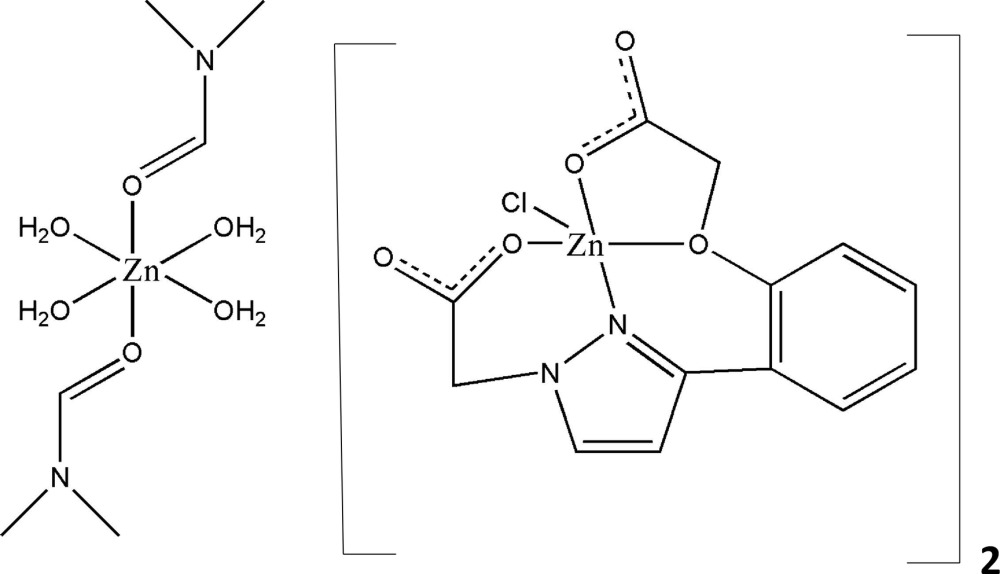


## Experimental

### 

#### Crystal data

[Zn(C_3_H_7_NO)_2_(H_2_O)_4_][Zn(C_13_H_10_N_2_O_5_)Cl]_2_
*M* *_r_* = 1033.79
Triclinic, 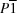

*a* = 8.0040 (16) Å
*b* = 8.7276 (17) Å
*c* = 15.782 (3) Å
α = 90.06 (3)°
β = 101.22 (3)°
γ = 107.95 (3)°
*V* = 1026.6 (3) Å^3^
*Z* = 1
Mo *K*α radiation
μ = 1.95 mm^−1^
*T* = 291 K
0.25 × 0.22 × 0.21 mm

#### Data collection

Rigaku Mercury diffractometer
Absorption correction: multi-scan (*ABSCOR*; Higashi, 1995 ▶) *T*_min_ = 0.642, *T*_max_ = 0.686
10740 measured reflections
4704 independent reflections
3632 reflections with *I* > 2σ(*I*)
*R*_int_ = 0.051

#### Refinement

*R*[*F* ^2^ > 2σ(*F* ^2^)] = 0.053
*wR*(*F* ^2^) = 0.168
*S* = 1.07
4704 reflections
265 parameters
23 restraints
H-atom parameters constrained
Δρ_max_ = 1.99 e Å^−3^
Δρ_min_ = −0.73 e Å^−3^

### 

Data collection: *CrystalClear* (Rigaku/MSC, 2001 ▶); cell refinement: *CrystalClear*; data reduction: *CrystalClear*; program(s) used to solve structure: *SHELXS97* (Sheldrick, 2008 ▶); program(s) used to refine structure: *SHELXL97* (Sheldrick, 2008 ▶); molecular graphics: *SHELXTL* (Sheldrick, 2008 ▶) and *CrystalStructure* (Rigaku/MSC, 2004 ▶); software used to prepare material for publication: *SHELXL97*.

## Supplementary Material

Crystal structure: contains datablock(s) I, global. DOI: 10.1107/S1600536812023045/su2397sup1.cif

Structure factors: contains datablock(s) I. DOI: 10.1107/S1600536812023045/su2397Isup2.hkl

Additional supplementary materials:  crystallographic information; 3D view; checkCIF report

## Figures and Tables

**Table 1:** Hydrogen-bond geometry (Å, °)

*D*—H⋯*A*	*D*—H	H⋯*A*	*D*⋯*A*	*D*—H⋯*A*
O7—H7*B*⋯O3^i^	0.96	1.83	2.710 (5)	151
O8—H8*B*⋯O2^ii^	0.96	2.21	2.941 (5)	132
O8—H8*C*⋯O5^iii^	0.96	1.91	2.715 (5)	140
C2—H2*A*⋯Cl1^iv^	0.93	2.79	3.668 (5)	159
C10—H10*B*⋯O5^v^	0.97	2.59	3.511 (6)	159

Symmetry codes: (i) 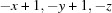; (ii) 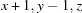; (iii) 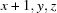; (iv) 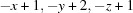; (v) 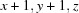.

**Fig. 1.**
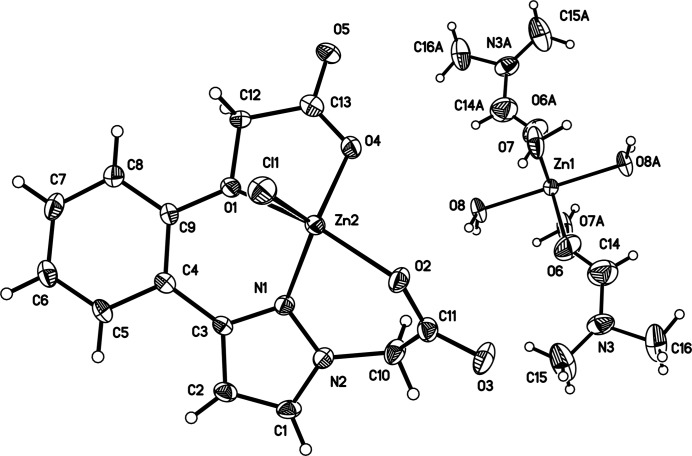
The molecular structure for title compound, showing the atom numbering. Displacement ellipsoids are drawn at the 30% probability level [symmetry code: (a) = -x+2, -y, -z].

**Fig. 2.**
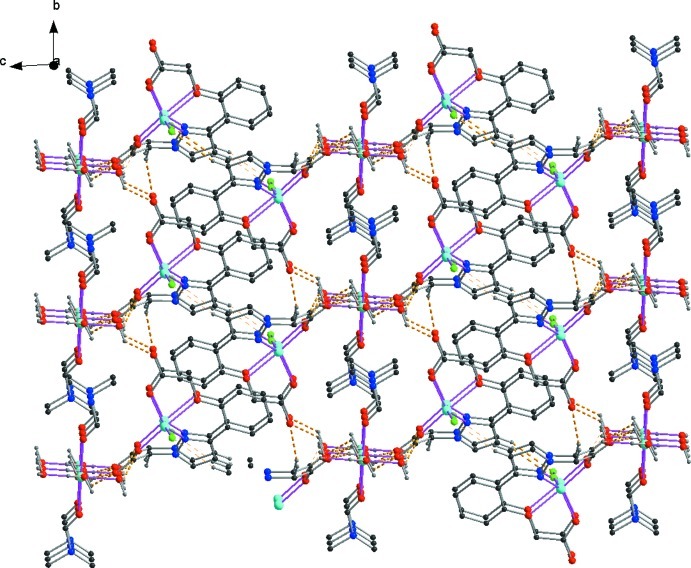
A view along the a axis of the crystal packing of the title compound, with the hydrogen bonds shown as dashed lines - see Table 1 for details.

## supplementary crystallographic information

### Comment

Coordination compounds containing a pyrazole group have been the subject of an intense research effort in recent years, owing to their unique structures and their potential applications in advanced materials (Su *et al.*, 2000; Tong *et al.*, 2003). Pyrazole-derived ligands, such as [3-(2-Carboxymethoxy-phenyl)-pyrazol-1-yl]-acetic acid, have been the subject of limited studies with metal ions, and no coordination complexes have been reported to date. The title ligand comprises N atoms of the pyrazole group and O atoms of the carboxylate, which should display flexible coordination behaviour.

The molecular structure of the title compound is shown in Fig. 1. In the dication atom Zn1 is located on an inversion center. It is coordinated by four O atoms from four water molecules and two O atoms from two DMF molecules, forming a slightly distorted octahedron.

In the monoanion atom Zn2 is coordinated by one nitrogen atom (N1) from the pyrazolyl, three oxygen atoms (O1, O2, O4) from the carboxylate groups and one chlorine atom, leading to a highly distorted trigonal bipyramidal geometry [the τ factor is 0.69; for perfect SP τ = 0, while for perfect TBP τ = 1.0 (Addison *et al.*, 1984). The ligand is chelated to the zinc(II) atom through a carboxylate bridge (O2, O4), a phenoxide group (O1) and a pyrazole nitrogen atom (N1) to form one five-membered and two six-membered chelate rings.

In the crystal, the ions are linked by O-H···O hydrogen-bonds, to form a two-dimensional network lying parallel to (001). There are also C-H···O and C-H···Cl interactions present leading to the formation of a three-dimensional structure (Table 1 and Fig. 2).

### Experimental

The title compound was synthesized by the reaction of [3-(2-Carboxymethoxy-phenyl)-pyrazol-1-yl]-acetic acid (0.0552 g, 0.2 mmol) and ZnCl_2_^.^4H_2_O (0.0209 mg, 0.1 mmol) in DMF (5 mL). The mixture was sealed in a 25 ml Teflon lined stainless steel container, which was heated at 363 K for 48 h and then cooled to room temperature. Colourless block-like crystals were obtained in *ca*. 64% yield based on Zn. Analysis, found: C, 28.55; H, 4.08; N, 24.57%. calculated: C, 28.57; H, 4.04; N, 24.55%.

### Refinement

The H-atoms were included in calculated positions and treated as riding atoms: O-H = 0.96 Å; C-H = 0.93, 0.96 and 0.97 Å for CH, CH_3_ and CH_2_ H-atoms, respectively, with U_iso_(H) = k × U_eq_(O,C), where k = 1.5 for OH and CH_3_ H-atoms and = 1.2 for other H-atoms.

### Crystal data

**Table:**

[Zn(C_3_H_7_NO)_2_(H_2_O)_4_][Zn(C_13_H_10_N_2_O_5_)Cl]_2_	*Z* = 1
*M**_r_* = 1033.79	*F*(000) = 528
Triclinic, *P*1	*D*_x_ = 1.672 Mg m^−^^3^
Hall symbol: -P 1	Mo *K*α radiation, λ = 0.71073 Å
*a* = 8.0040 (16) Å	Cell parameters from 10740 reflections
*b* = 8.7276 (17) Å	θ = 3.1–27.5°
*c* = 15.782 (3) Å	µ = 1.95 mm^−^^1^
α = 90.06 (3)°	*T* = 291 K
β = 101.22 (3)°	Block, colourless
γ = 107.95 (3)°	0.25 × 0.22 × 0.21 mm
*V* = 1026.6 (3) Å^3^	

### Data collection

**Table:**

Rigaku Mercury diffractometer	4704 independent reflections
Radiation source: fine-focus sealed tube	3632 reflections with *I* > 2σ(*I*)
Graphite monochromator	*R*_int_ = 0.051
ω scans	θ_max_ = 27.5°, θ_min_ = 3.1°
Absorption correction: multi-scan (*ABSCOR*; Higashi, 1995)	*h* = −10→10
*T*_min_ = 0.642, *T*_max_ = 0.686	*k* = −11→11
10740 measured reflections	*l* = −20→20

### Refinement

**Table:**

Refinement on *F*^2^	Primary atom site location: structure-invariant direct methods
Least-squares matrix: full	Secondary atom site location: difference Fourier map
*R*[*F*^2^ > 2σ(*F*^2^)] = 0.053	Hydrogen site location: inferred from neighbouring sites
*wR*(*F*^2^) = 0.168	H-atom parameters constrained
*S* = 1.07	*w* = 1/[σ^2^(*F*_o_^2^) + (0.1*P*)^2^] where *P* = (*F*_o_^2^ + 2*F*_c_^2^)/3
4704 reflections	(Δ/σ)_max_ < 0.001
265 parameters	Δρ_max_ = 1.99 e Å^−^^3^
23 restraints	Δρ_min_ = −0.73 e Å^−^^3^

### Special details

**Table:**

Geometry. Bond distances, angles etc. have been calculated using the rounded fractional coordinates. All su's are estimated from the variances of the (full) variance-covariance matrix. The cell esds are taken into account in the estimation of distances, angles and torsion angles
Refinement. Refinement of *F*^2^ against ALL reflections. The weighted *R*-factor *wR* and goodness of fit *S* are based on *F*^2^, conventional *R*-factors *R* are based on *F*, with *F* set to zero for negative *F*^2^. The threshold expression of *F*^2^ > σ(*F*^2^) is used only for calculating *R*-factors(gt) *etc*. and is not relevant to the choice of reflections for refinement. *R*-factors based on *F*^2^ are statistically about twice as large as those based on *F*, and *R*- factors based on ALL data will be even larger.

### Fractional atomic coordinates and isotropic or equivalent isotropic displacement parameters (Å^2^)

**Table:**

	*x*	*y*	*z*	*U*_iso_*/*U*_eq_	
Zn2	0.28061 (6)	0.71444 (5)	0.29770 (3)	0.0271 (2)	
Cl1	0.04523 (14)	0.78125 (14)	0.32248 (8)	0.0415 (4)	
O1	0.2909 (4)	0.5528 (3)	0.41252 (18)	0.0343 (9)	
O2	0.3455 (4)	0.8648 (4)	0.20287 (18)	0.0371 (9)	
O3	0.5276 (4)	1.0071 (5)	0.1224 (2)	0.0509 (13)	
O4	0.2013 (4)	0.4924 (3)	0.24461 (19)	0.0365 (9)	
O5	0.0503 (4)	0.2338 (4)	0.2575 (2)	0.0459 (11)	
N1	0.5313 (4)	0.8109 (4)	0.3703 (2)	0.0283 (10)	
N2	0.6622 (4)	0.9126 (4)	0.3366 (2)	0.0301 (10)	
C1	0.7964 (5)	1.0017 (5)	0.3987 (3)	0.0361 (12)	
C2	0.7530 (6)	0.9617 (5)	0.4766 (3)	0.0350 (12)	
C3	0.5845 (5)	0.8392 (4)	0.4578 (2)	0.0249 (11)	
C4	0.4840 (5)	0.7501 (5)	0.5203 (3)	0.0277 (11)	
C5	0.5382 (6)	0.8039 (5)	0.6081 (3)	0.0336 (12)	
C6	0.4588 (7)	0.7199 (6)	0.6715 (3)	0.0413 (16)	
C7	0.3213 (7)	0.5759 (6)	0.6478 (3)	0.0432 (16)	
C8	0.2595 (6)	0.5178 (5)	0.5621 (3)	0.0348 (12)	
C9	0.3411 (5)	0.6049 (5)	0.4985 (2)	0.0273 (11)	
C10	0.6618 (6)	0.9009 (6)	0.2440 (3)	0.0398 (14)	
C11	0.4999 (5)	0.9281 (5)	0.1858 (3)	0.0339 (12)	
C12	0.1551 (5)	0.4021 (4)	0.3845 (3)	0.0281 (11)	
C13	0.1363 (6)	0.3744 (5)	0.2887 (3)	0.0348 (12)	
Zn1	1.00000	0.00000	0.00000	0.0325 (2)	
O6	1.0708 (8)	0.2476 (5)	−0.0070 (4)	0.094 (2)	
O7	0.7437 (5)	−0.0088 (7)	0.0101 (2)	0.0810 (19)	
O8	1.0867 (5)	0.0342 (4)	0.13520 (19)	0.0494 (11)	
N3	1.2254 (6)	0.4929 (5)	−0.0348 (3)	0.0660 (18)	
C14	1.1206 (6)	0.3353 (5)	−0.0562 (3)	0.101 (3)	
C15	1.3142 (14)	0.5932 (12)	0.0394 (5)	0.117 (4)	
C16	1.2371 (14)	0.5717 (13)	−0.1167 (5)	0.127 (5)	
H1A	0.90130	1.07770	0.39000	0.0430*	
H2A	0.82010	1.00570	0.53090	0.0420*	
H5A	0.63130	0.90010	0.62420	0.0400*	
H6A	0.49700	0.75920	0.72910	0.0500*	
H7A	0.26940	0.51700	0.69030	0.0520*	
H8A	0.16510	0.42230	0.54690	0.0420*	
H10A	0.66620	0.79470	0.22870	0.0480*	
H10B	0.76960	0.97980	0.23300	0.0480*	
H12A	0.04210	0.40420	0.39730	0.0340*	
H12B	0.18820	0.31550	0.41460	0.0340*	
H7B	0.66940	−0.02460	−0.04690	0.1210*	
H7C	0.74740	0.09090	0.03730	0.1210*	
H8B	1.21120	0.04140	0.15010	0.0740*	
H8C	1.07190	0.13250	0.15470	0.0740*	
H14A	1.08940	0.28160	−0.11290	0.1510*	
H15A	1.30250	0.53170	0.08960	0.1750*	
H15B	1.26260	0.67830	0.04230	0.1750*	
H15C	1.43890	0.63880	0.03760	0.1750*	
H16A	1.17280	0.49420	−0.16430	0.1900*	
H16B	1.36070	0.61470	−0.12100	0.1900*	
H16C	1.18550	0.65770	−0.11810	0.1900*	

### Atomic displacement parameters (Å^2^)

**Table:**

	*U*^11^	*U*^22^	*U*^33^	*U*^12^	*U*^13^	*U*^23^
Zn2	0.0249 (3)	0.0290 (3)	0.0234 (3)	0.0051 (2)	0.0009 (2)	0.0017 (2)
Cl1	0.0314 (5)	0.0477 (7)	0.0473 (7)	0.0147 (5)	0.0092 (5)	−0.0004 (5)
O1	0.0401 (16)	0.0295 (15)	0.0255 (14)	0.0018 (12)	0.0031 (12)	0.0017 (12)
O2	0.0267 (14)	0.0531 (19)	0.0271 (15)	0.0065 (13)	0.0048 (12)	0.0146 (14)
O3	0.0330 (17)	0.083 (3)	0.0349 (18)	0.0130 (17)	0.0115 (14)	0.0271 (17)
O4	0.0455 (17)	0.0321 (16)	0.0290 (15)	0.0076 (13)	0.0084 (13)	−0.0017 (12)
O5	0.0502 (19)	0.0320 (17)	0.0456 (19)	−0.0007 (14)	0.0089 (15)	−0.0144 (14)
N1	0.0214 (15)	0.0339 (18)	0.0270 (17)	0.0061 (14)	0.0030 (13)	0.0041 (14)
N2	0.0237 (16)	0.0361 (19)	0.0286 (18)	0.0079 (14)	0.0033 (14)	0.0075 (14)
C1	0.025 (2)	0.033 (2)	0.040 (2)	−0.0009 (17)	−0.0014 (18)	0.0049 (19)
C2	0.029 (2)	0.034 (2)	0.034 (2)	0.0038 (17)	−0.0023 (17)	−0.0019 (18)
C3	0.0261 (18)	0.0230 (18)	0.0244 (19)	0.0099 (15)	−0.0013 (15)	0.0002 (15)
C4	0.0262 (19)	0.029 (2)	0.028 (2)	0.0126 (16)	−0.0004 (16)	0.0002 (16)
C5	0.039 (2)	0.036 (2)	0.024 (2)	0.0140 (18)	−0.0012 (17)	−0.0048 (17)
C6	0.046 (3)	0.053 (3)	0.026 (2)	0.019 (2)	0.0041 (19)	−0.004 (2)
C7	0.046 (3)	0.058 (3)	0.030 (2)	0.017 (2)	0.017 (2)	0.013 (2)
C8	0.035 (2)	0.037 (2)	0.031 (2)	0.0090 (19)	0.0075 (18)	0.0018 (18)
C9	0.029 (2)	0.032 (2)	0.0230 (19)	0.0133 (17)	0.0040 (16)	0.0000 (16)
C10	0.029 (2)	0.056 (3)	0.035 (2)	0.012 (2)	0.0103 (18)	0.013 (2)
C11	0.029 (2)	0.045 (2)	0.026 (2)	0.0102 (19)	0.0038 (17)	0.0036 (18)
C12	0.0272 (19)	0.0220 (19)	0.032 (2)	0.0034 (15)	0.0057 (16)	0.0013 (16)
C13	0.031 (2)	0.034 (2)	0.040 (2)	0.0138 (18)	0.0027 (18)	−0.0043 (19)
Zn1	0.0342 (4)	0.0339 (4)	0.0301 (4)	0.0116 (3)	0.0071 (3)	0.0034 (3)
O6	0.133 (4)	0.050 (3)	0.098 (4)	0.022 (3)	0.030 (3)	0.031 (2)
O7	0.050 (2)	0.177 (5)	0.0319 (19)	0.058 (3)	0.0093 (17)	0.007 (2)
O8	0.070 (2)	0.061 (2)	0.0268 (16)	0.0414 (19)	−0.0013 (15)	−0.0040 (15)
N3	0.073 (3)	0.039 (2)	0.090 (4)	0.014 (2)	0.032 (3)	0.002 (2)
C14	0.116 (5)	0.069 (4)	0.113 (5)	0.017 (4)	0.031 (4)	0.004 (4)
C15	0.168 (8)	0.130 (7)	0.066 (5)	0.082 (6)	0.001 (5)	−0.018 (5)
C16	0.157 (9)	0.163 (9)	0.052 (5)	0.049 (7)	0.005 (5)	0.022 (5)

### Geometric parameters (Å, º)

**Table:**

Zn2—Cl1	2.2373 (14)	C1—C2	1.359 (7)
Zn2—O1	2.302 (3)	C2—C3	1.416 (6)
Zn2—O2	2.029 (3)	C3—C4	1.466 (6)
Zn2—O4	1.973 (3)	C4—C5	1.403 (7)
Zn2—N1	2.027 (3)	C4—C9	1.409 (6)
Zn1—O6	2.067 (4)	C5—C6	1.380 (7)
Zn1—O7	2.066 (4)	C6—C7	1.385 (7)
Zn1—O8	2.102 (3)	C7—C8	1.384 (7)
Zn1—O6^i^	2.067 (4)	C8—C9	1.401 (6)
Zn1—O7^i^	2.066 (4)	C10—C11	1.519 (7)
Zn1—O8^i^	2.102 (3)	C12—C13	1.502 (7)
O1—C9	1.372 (4)	C1—H1A	0.9300
O1—C12	1.427 (5)	C2—H2A	0.9300
O2—C11	1.271 (5)	C5—H5A	0.9300
O3—C11	1.233 (6)	C6—H6A	0.9300
O4—C13	1.276 (5)	C7—H7A	0.9300
O5—C13	1.252 (5)	C8—H8A	0.9300
O6—C14	1.132 (7)	C10—H10B	0.9700
O7—H7B	0.9600	C10—H10A	0.9700
O7—H7C	0.9600	C12—H12B	0.9700
O8—H8C	0.9600	C12—H12A	0.9700
O8—H8B	0.9600	C14—H14A	0.9600
N1—N2	1.347 (5)	C15—H15A	0.9600
N1—C3	1.362 (4)	C15—H15B	0.9600
N2—C1	1.339 (6)	C15—H15C	0.9600
N2—C10	1.464 (6)	C16—H16A	0.9600
N3—C15	1.390 (10)	C16—H16B	0.9600
N3—C16	1.470 (10)	C16—H16C	0.9600
N3—C14	1.374 (6)		
			
Cl1—Zn2—O1	94.96 (9)	C3—C4—C5	119.3 (4)
Cl1—Zn2—O2	99.10 (11)	C4—C5—C6	122.6 (4)
Cl1—Zn2—O4	110.29 (11)	C5—C6—C7	118.8 (4)
Cl1—Zn2—N1	122.65 (10)	C6—C7—C8	121.3 (4)
O1—Zn2—O2	164.05 (13)	C7—C8—C9	119.1 (4)
O1—Zn2—O4	74.99 (11)	O1—C9—C4	116.2 (3)
O1—Zn2—N1	75.37 (12)	O1—C9—C8	122.5 (4)
O2—Zn2—O4	106.75 (13)	C4—C9—C8	121.2 (3)
O2—Zn2—N1	90.64 (13)	N2—C10—C11	114.2 (4)
O4—Zn2—N1	120.51 (14)	O2—C11—C10	119.1 (4)
O7—Zn1—O7^i^	180.00	O2—C11—O3	124.1 (4)
O7—Zn1—O8^i^	89.06 (15)	O3—C11—C10	116.8 (4)
O6^i^—Zn1—O8	90.8 (2)	O1—C12—C13	108.3 (3)
O7^i^—Zn1—O8	89.06 (15)	O4—C13—O5	124.6 (4)
O8—Zn1—O8^i^	180.00	O5—C13—C12	115.8 (4)
O6^i^—Zn1—O7^i^	90.4 (2)	O4—C13—C12	119.6 (4)
O6^i^—Zn1—O8^i^	89.2 (2)	C2—C1—H1A	126.00
O7^i^—Zn1—O8^i^	90.94 (15)	N2—C1—H1A	126.00
O7—Zn1—O8	90.94 (15)	C1—C2—H2A	127.00
O6^i^—Zn1—O7	89.6 (2)	C3—C2—H2A	127.00
O6—Zn1—O7	90.4 (2)	C6—C5—H5A	119.00
O6—Zn1—O8	89.2 (2)	C4—C5—H5A	119.00
O6—Zn1—O6^i^	180.00	C5—C6—H6A	121.00
O6—Zn1—O7^i^	89.6 (2)	C7—C6—H6A	121.00
O6—Zn1—O8^i^	90.8 (2)	C8—C7—H7A	119.00
Zn2—O1—C12	106.2 (2)	C6—C7—H7A	119.00
C9—O1—C12	120.6 (3)	C7—C8—H8A	120.00
Zn2—O1—C9	125.9 (2)	C9—C8—H8A	120.00
Zn2—O2—C11	127.5 (3)	N2—C10—H10A	109.00
Zn2—O4—C13	119.3 (3)	C11—C10—H10B	109.00
Zn1—O6—C14	135.3 (5)	H10A—C10—H10B	108.00
H7B—O7—H7C	109.00	N2—C10—H10B	109.00
Zn1—O7—H7C	110.00	C11—C10—H10A	109.00
Zn1—O7—H7B	109.00	C13—C12—H12A	110.00
H8B—O8—H8C	109.00	O1—C12—H12B	110.00
Zn1—O8—H8B	109.00	O1—C12—H12A	110.00
Zn1—O8—H8C	109.00	C13—C12—H12B	110.00
N2—N1—C3	106.0 (3)	H12A—C12—H12B	108.00
Zn2—N1—C3	129.2 (3)	O6—C14—N3	123.6 (5)
Zn2—N1—N2	120.7 (2)	O6—C14—H14A	111.00
N1—N2—C1	111.5 (3)	N3—C14—H14A	125.00
N1—N2—C10	121.0 (3)	N3—C15—H15A	109.00
C1—N2—C10	126.8 (4)	N3—C15—H15B	110.00
C14—N3—C16	106.6 (5)	N3—C15—H15C	109.00
C14—N3—C15	138.4 (6)	H15A—C15—H15B	109.00
C15—N3—C16	115.0 (6)	H15A—C15—H15C	109.00
N2—C1—C2	108.2 (4)	H15B—C15—H15C	109.00
C1—C2—C3	105.7 (4)	N3—C16—H16A	109.00
N1—C3—C2	108.6 (3)	N3—C16—H16B	109.00
N1—C3—C4	124.3 (3)	N3—C16—H16C	109.00
C2—C3—C4	127.0 (3)	H16A—C16—H16B	110.00
C3—C4—C9	123.7 (4)	H16A—C16—H16C	110.00
C5—C4—C9	116.9 (4)	H16B—C16—H16C	109.00
			
Cl1—Zn2—O1—C9	−69.9 (3)	Zn2—N1—N2—C10	−29.1 (5)
Cl1—Zn2—O1—C12	79.9 (2)	C3—N1—N2—C1	1.0 (4)
O4—Zn2—O1—C9	−179.6 (4)	Zn2—N1—N2—C1	160.1 (3)
O4—Zn2—O1—C12	−29.8 (2)	N2—N1—C3—C4	−177.7 (4)
N1—Zn2—O1—C9	52.6 (3)	C3—N1—N2—C10	171.8 (4)
N1—Zn2—O1—C12	−157.7 (3)	Zn2—N1—C3—C2	−156.8 (3)
Cl1—Zn2—O2—C11	150.2 (3)	Zn2—N1—C3—C4	25.6 (6)
O4—Zn2—O2—C11	−95.3 (4)	N2—N1—C3—C2	−0.1 (4)
N1—Zn2—O2—C11	27.0 (4)	N1—N2—C10—C11	61.6 (5)
Cl1—Zn2—O4—C13	−66.6 (4)	C1—N2—C10—C11	−129.1 (4)
O1—Zn2—O4—C13	23.2 (3)	C10—N2—C1—C2	−171.6 (4)
O2—Zn2—O4—C13	−173.3 (3)	N1—N2—C1—C2	−1.4 (5)
N1—Zn2—O4—C13	85.7 (4)	C15—N3—C14—O6	6.5 (13)
Cl1—Zn2—N1—N2	−111.0 (3)	C16—N3—C14—O6	−171.4 (7)
Cl1—Zn2—N1—C3	42.7 (4)	N2—C1—C2—C3	1.3 (5)
O1—Zn2—N1—N2	162.5 (3)	C1—C2—C3—N1	−0.7 (5)
O1—Zn2—N1—C3	−43.8 (3)	C1—C2—C3—C4	176.8 (4)
O2—Zn2—N1—N2	−9.8 (3)	N1—C3—C4—C5	−171.2 (4)
O2—Zn2—N1—C3	143.9 (3)	N1—C3—C4—C9	13.3 (6)
O4—Zn2—N1—N2	100.2 (3)	C2—C3—C4—C5	11.7 (7)
O4—Zn2—N1—C3	−106.1 (3)	C2—C3—C4—C9	−163.8 (4)
O7^i^—Zn1—O6—C14	60.2 (8)	C3—C4—C9—O1	−3.7 (6)
O8^i^—Zn1—O6—C14	−30.8 (8)	C3—C4—C5—C6	−175.1 (5)
O7—Zn1—O6—C14	−119.8 (8)	C9—C4—C5—C6	0.7 (7)
O8—Zn1—O6—C14	149.3 (8)	C5—C4—C9—C8	−0.9 (6)
C12—O1—C9—C4	176.1 (4)	C3—C4—C9—C8	174.6 (4)
C9—O1—C12—C13	−177.2 (4)	C5—C4—C9—O1	−179.3 (4)
C12—O1—C9—C8	−2.2 (6)	C4—C5—C6—C7	0.7 (8)
Zn2—O1—C12—C13	31.1 (4)	C5—C6—C7—C8	−1.8 (8)
Zn2—O1—C9—C4	−38.1 (5)	C6—C7—C8—C9	1.6 (8)
Zn2—O1—C9—C8	143.6 (4)	C7—C8—C9—O1	178.1 (4)
Zn2—O2—C11—O3	173.3 (3)	C7—C8—C9—C4	−0.2 (7)
Zn2—O2—C11—C10	−4.1 (6)	N2—C10—C11—O2	−42.3 (6)
Zn2—O4—C13—O5	165.2 (4)	N2—C10—C11—O3	140.2 (4)
Zn2—O4—C13—C12	−12.3 (6)	O1—C12—C13—O4	−16.5 (6)
Zn1—O6—C14—N3	−152.5 (5)	O1—C12—C13—O5	165.7 (4)

Symmetry code: (i) −*x*+2, −*y*, −*z*.

### Hydrogen-bond geometry (Å, º)

**Table:**

*D*—H···*A*	*D*—H	H···*A*	*D*···*A*	*D*—H···*A*
O7—H7*B*···O3^ii^	0.96	1.83	2.710 (5)	151
O8—H8*B*···O2^iii^	0.96	2.21	2.941 (5)	132
O8—H8*C*···O5^iv^	0.96	1.91	2.715 (5)	140
C2—H2*A*···Cl1^v^	0.93	2.79	3.668 (5)	159
C10—H10*B*···O5^vi^	0.97	2.59	3.511 (6)	159

Symmetry codes: (ii) −*x*+1, −*y*+1, −*z*; (iii) *x*+1, *y*−1, *z*; (iv) *x*+1, *y*, *z*; (v) −*x*+1, −*y*+2, −*z*+1; (vi) *x*+1, *y*+1, *z*.
